# Meta-Analysis of RNA-Seq Data Identifies Differentially Expressed Genes in Skeletal Muscle Between Obese and Normal Weight Individuals

**DOI:** 10.3390/ijms27062677

**Published:** 2026-03-15

**Authors:** Yuhao Wang, Han Li, Yixuan Li, Wen Kong, Yuming Li

**Affiliations:** Department of Endocrinology, Union Hospital, Tongji Medical College, Huazhong University of Science and Technology, Wuhan 430022, China; u201710310@hust.edu.cn (Y.W.); phoebepagelee@163.com (H.L.); 15225503586@163.com (Y.L.)

**Keywords:** obesity, skeletal muscle, RNA-seq, meta-analysis, insulin resistance

## Abstract

Obesity disrupts skeletal muscle metabolism through insulin resistance, oxidative stress, and ectopic fat deposition, yet transcriptomic findings across individual studies remain inconsistent. We performed a meta-analysis of four independent RNA sequencing (RNA-seq) studies of human vastus lateralis muscle, comparing 29 individuals with obesity (body mass index (BMI) ≥ 30 kg/m^2^) and 23 with normal weight. Differential expression was analyzed using DESeq2, with age and sex included as covariates in studies where individual-level data were available. Study-level results were integrated using the direction-aware inverse normal method (weighted Stouffer). Between-study heterogeneity was assessed by gene-level I^2^ statistics derived from random-effects meta-analysis of log_2_ fold changes. Functional annotation was performed with Gene Ontology and Kyoto Encyclopedia of Genes and Genomes (KEGG) pathway analyses. The weighted Stouffer method identified 2136 differentially expressed genes (DEGs) (adjusted *p* < 0.05), comprising 1028 upregulated and 1108 downregulated genes, of which 674 (31.6%) were detected only through the meta-analysis. Three genes—*PHLDA3* (down), *CNKSR2* (down), and *SFRP4* (up)—were significant in every individual study and in the combined analysis. Downregulated DEGs were enriched in cytoplasmic translation, ribosomal structure, and oxidative phosphorylation, whereas upregulated DEGs were associated with extracellular matrix organization and the focal adhesion pathway. This RNA-seq meta-analysis of skeletal muscle in obesity identifies robust DEGs and dysregulated pathways, providing candidate targets for future mechanistic and translational research.

## 1. Introduction

Obesity is characterized by excessive or abnormal fat accumulation that poses a significant health risk [1]. The World Health Organization (WHO) defines overweight as a body mass index (BMI) ≥ 25 and obesity as a BMI ≥ 30. Recent findings from the Global Burden of Disease Obesity Collaborators reveal that 603.7 million adults worldwide are affected by obesity, with elevated BMI contributing to 4 million deaths annually [2,3]. Obesity predisposes individuals to an elevated risk of a spectrum of diseases, including type 2 diabetes, cardiovascular disorders, and cancers [4]. Obesity is a key driver of skeletal muscle dysfunction, disrupting muscle metabolism via mechanisms such as insulin resistance, mitochondrial dysfunction, and oxidative stress [5,6,7]. Insulin resistance markedly reduces metabolic efficiency in peripheral tissues—including skeletal muscle, liver, and adipose tissue—culminating in diminished glucose uptake by skeletal muscle and leading to metabolic disorders [8,9,10,11]. A hallmark of obesity is ectopic fat deposition, particularly the accumulation of intermuscular adipose tissue (IMAT), which severely compromises skeletal muscle function [12]. Physiologically, IMAT supports energy storage and microenvironmental homeostasis within muscle; however, excessive IMAT accumulation induces ectopic lipid infiltration into muscle fibers, contributing to significant muscle dysfunction [13,14,15]. Sarcopenic obesity (SO), a pathological state arising from the interplay of obesity and sarcopenia, further intensifies risks of metabolic disorders and functional decline [16,17]. Ectopic fat infiltration reduces muscle strength, exacerbating vulnerability to falls, fractures, and mobility impairments [18,19]. Therefore, elucidating the mechanistic interactions between obesity and skeletal muscle dysfunction is imperative for the development of precision-based interventions aimed at mitigating the metabolic and functional consequences of obesity.

RNA sequencing (RNA-seq) is a powerful used tool in transcriptomics, allowing identification of gene expression changes associated with obesity in skeletal muscle. Numerous studies leveraging RNA-seq have identified differentially expressed genes (DEGs) in skeletal muscle tissues from lean and obese individuals, with commonly reported DEGs including *F13A1*, *GDNF*, and *SLN* [20,21]. Nevertheless, variations in sample selection protocols, sequencing methodologies, and bioinformatic analyses have led to inconsistencies in the reported gene sets. Meta-analysis, a robust statistical method for synthesizing data across multiple studies, offers a solution to resolve these discrepancies [22]. Herein, we performed a meta-analysis of four RNA-seq datasets to identify robust DEGs in skeletal muscle between individuals with obesity and those who are lean.

## 2. Results

### 2.1. RNA-Seq Study Data Sources

A total of three studies [23,24,25] were included from PubMed, and one study was identified from the GEO database. The details of the four RNA-seq studies (GSE231509, GSE185957, GSE137631, and GSE196387) are presented in Table 1.

A total of 29 individuals with obesity and 23 individuals with normal body weight were selected for inclusion. Forty-eight samples were excluded due to factors such as exercise, metabolic surgery, or the presence of type 2 diabetes (T2DM). The demographic characteristics are summarized in Table 2. Across studies, mean BMI in the non-obese groups ranged from 22.3 to 24.0 kg/m^2^, whereas mean BMI in the obese groups ranged from 36.2 to 47.9 kg/m^2^. Mean age ranged from 34 to 43 years in the non-obese groups and from 37 to 46 years in the obese groups, except for GSE185957 where age was not reported. Sex distributions varied substantially by cohort.

### 2.2. Analysis of Differentially Expressed Genes

**Figure 1:**
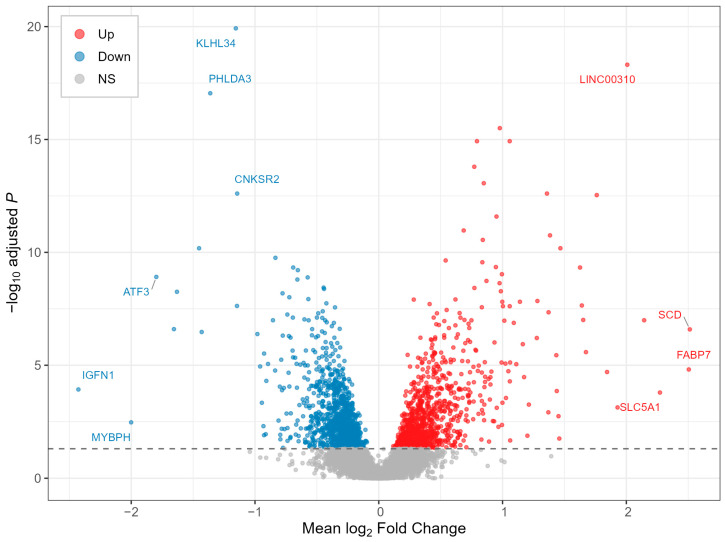
Volcano plot of meta-analysis results. Red and blue points indicate significantly upregulated and downregulated genes, respectively (adjusted *p*-value < 0.05).

The four individual studies identified 1013, 243, 832, and 1552 DEGs (adjusted *p*-value < 0.05), respectively. After low-expression filtering within each study, 12,208 genes were retained in all four datasets and entered the meta-analysis. Using a weighted Stouffer approach, we identified 2136 meta-analysis DEGs (adjusted *p*-value < 0.05), including 1028 genes with positive meta-analysis Z-scores and 1108 with negative meta-analysis Z-scores (Figure 1; Supplementary Table S1). Among the upregulated DEGs, *SCD* exhibited the highest mean log2 fold change (log2FC = 2.513), followed by *FABP7* (log2FC = 2.505). Among the downregulated DEGs, *IGFN1* showed the lowest mean log2FC (log2FC = −2.43).

**Figure 2:**
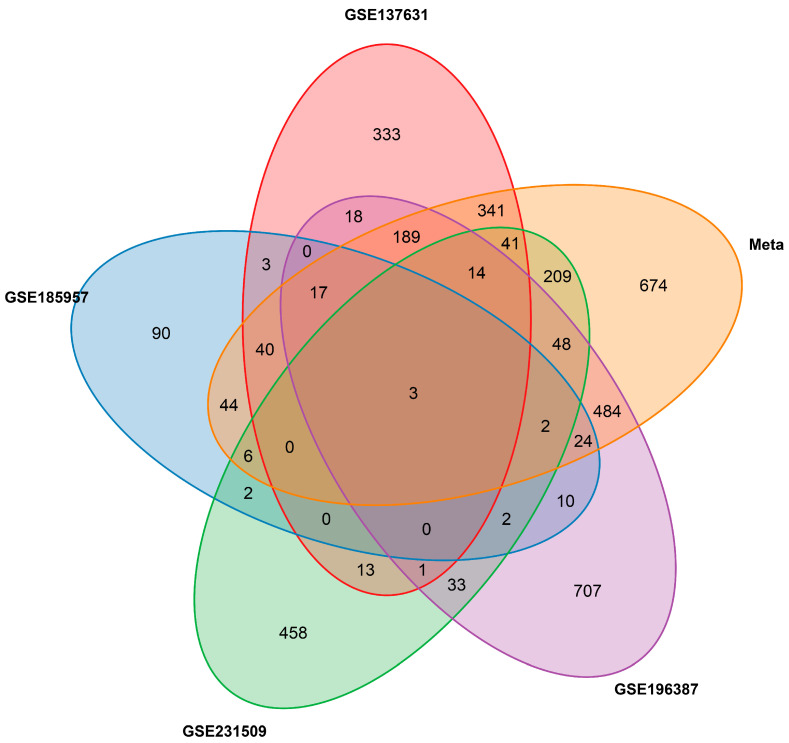
Venn diagram showing the overlap of differentially expressed genes among the four individual studies and the meta-analysis. Numbers indicate the count of DEGs unique to or shared between each set.

The Venn diagram illustrating the overlap of DEGs among the four individual studies and the meta-analysis is shown in Figure 2. The meta-analysis identified 674 DEGs (31.6%) not detected in any individual study, demonstrating the added value of combining data across studies. Only three genes—*CNKSR2*, *PHLDA3*, and *SFRP4*—were identified as common DEGs across all four individual studies and the meta-analysis.

Between-study heterogeneity was assessed using gene-level I^2^ from a random-effects meta-analysis of log2 fold changes. The median I^2^ was 37.4% (IQR: 0.0–65.4%) across all genes and 49.8% (IQR: 4.5–73.0%) among DEGs (Supplementary Figure S1).

### 2.3. Gene Ontology Enrichment Analysis

**Figure 3:**
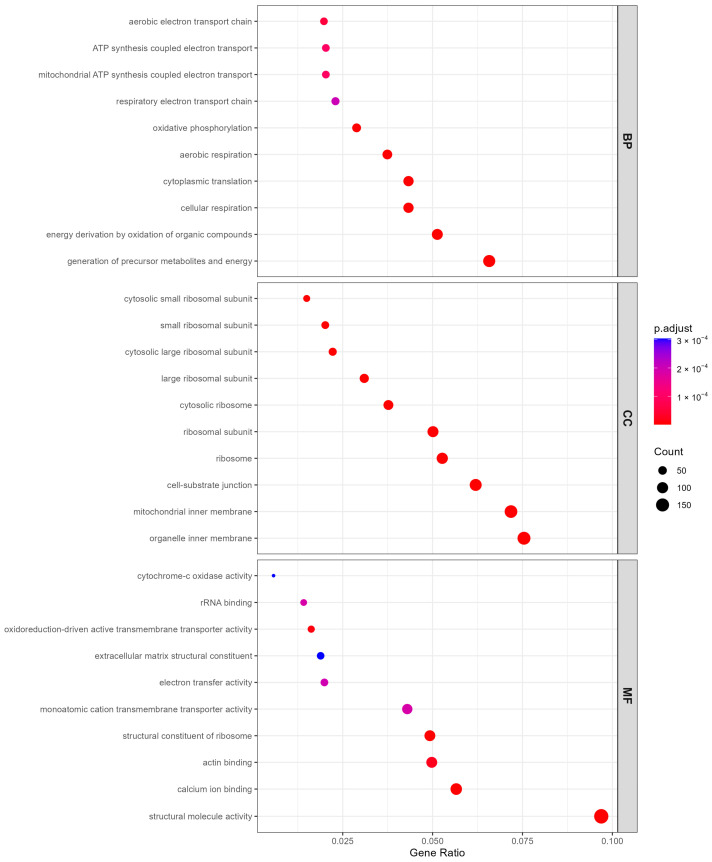
The top 10 biological process (BP), cellular component (CC), and molecular function (MF) terms in the significant Gene Ontology enrichment analysis.

Gene Ontology (GO) over-representation analysis (ORA) was performed for BP, CC and MF terms using the meta-analysis DEGs, with the 12,208 meta-analyzed genes as the background universe (Figure 3). In total, 50 BP terms, 71 CC terms and 41 MF terms reached significance at adjusted *p*-value < 0.05. Figure 3 shows the top enriched terms in each category (ranked by adjusted *p*-value). The enriched BP terms were dominated by mitochondrial respiration/oxidative phosphorylation and translation-related processes, while CC terms were primarily associated with ribosomal structures and mitochondrial inner membrane components. Consistently, MF terms highlighted structural constituent of ribosome/structural molecule activity and oxidoreductase-related activities.

### 2.4. Pathway Enrichment Analysis of Differentially Expressed Genes

**Figure 4:**
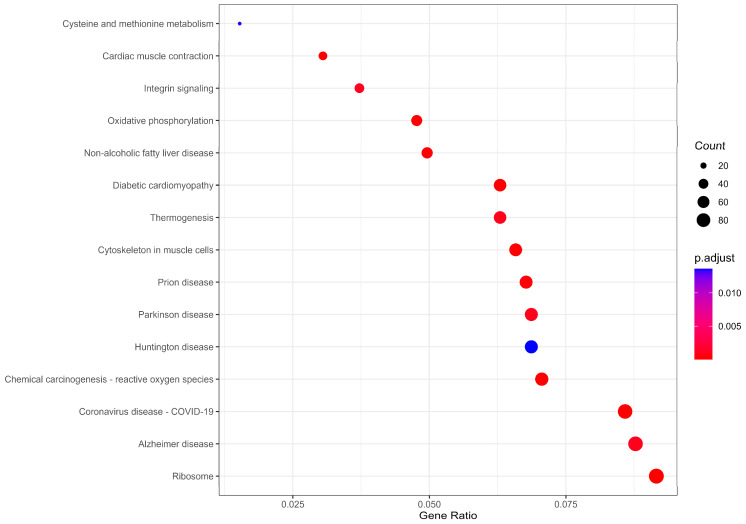
Dot plot of the top 15 enriched KEGG pathways. Dot size represents the number of DEGs in each pathway; color indicates the adjusted *p*-value.

**Figure 5:**
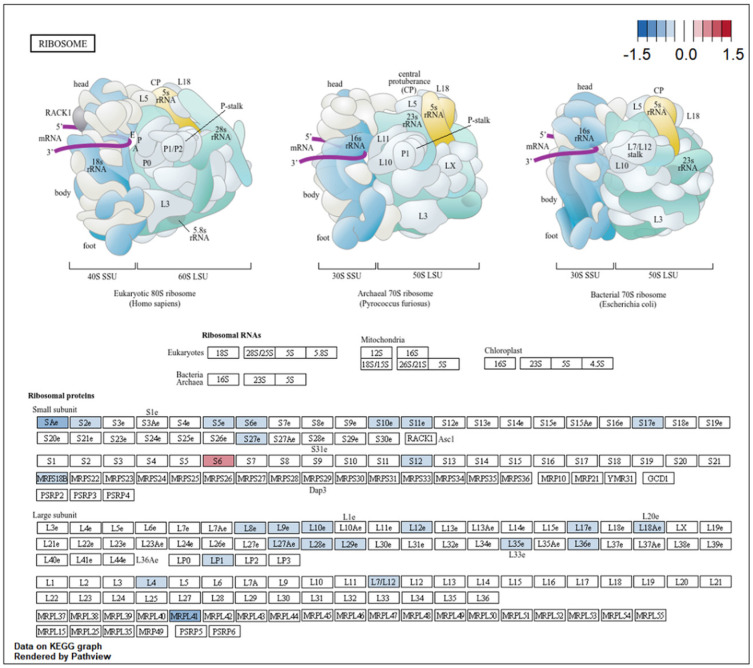
The pathway map of differentially expressed genes enriched in the ribosome pathway (KEGG: hsa03010). Rendered by Pathview (version 1.42.0).

**Figure 6:**
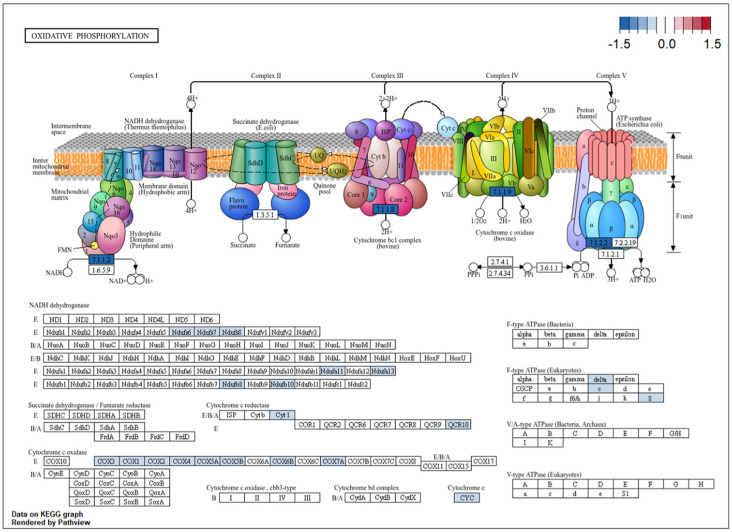
The pathway map of differentially expressed genes enriched in the oxidative phosphorylation pathway (KEGG: hsa00190). Rendered by Pathview (version 1.42.0).

KEGG pathway enrichment analysis identified 18 enriched pathways (Figure 4). The most significant signal was the ribosome pathway (hsa03010), which included 96 DEGs. Mapping these genes onto the KEGG ribosome diagram showed that many ribosome-related genes had negative mean log2FC values in the meta-analysis (Figure 5), consistent with broad shifts in translation-related transcriptional programs in obesity. Oxidative phosphorylation (hsa00190) was also significantly enriched and involved 50 DEGs distributed across multiple electron transport chain complexes (Figure 6). Many of these genes showed negative mean log2FC values, consistent with altered oxidative phosphorylation-related transcriptional programs in the skeletal muscle of individuals with obesity.

## 3. Discussion

In this study, we performed a transcriptome meta-analysis of skeletal muscle from individuals with obesity and normal weight by integrating four independent RNA-seq datasets. The meta-analysis identified 2136 DEGs (adjusted *p*-value < 0.05), including 1028 upregulated and 1108 downregulated genes, of which 674 (31.6%) were not detected in any individual study alone. Three genes—*PHLDA3*, *CNKSR2*, and *SFRP4*—were consistently significant across all four individual studies as well as the meta-analysis, representing robust candidates that persist despite inter-study heterogeneity.

The three shared genes map to distinct signaling pathways relevant to skeletal muscle metabolism and regeneration. *PHLDA3* was consistently downregulated across all four studies (mean log2FC = −1.42). *PHLDA3* is a p53 target that inhibits Akt signaling through competitive phosphoinositide binding [26], and has been linked to stress adaptation in pancreatic β cells [27]. In skeletal muscle, Akt is a key mediator of insulin-stimulated glucose uptake [11]; reduced *PHLDA3* may therefore relieve Akt inhibition as a compensatory response to obesity-related insulin resistance. *CNKSR2* was also downregulated (mean log2FC = −1.17). *CNKSR2* encodes a scaffold protein in the Ras/MAPK pathway that regulates signal amplitude and specificity [28]. MAPK/ERK cascades coordinate postnatal myogenesis, including satellite cell activation, myoblast proliferation, and myofiber hypertrophy [29]. Reduced *CNKSR2* expression may impair MAPK signaling efficiency in skeletal muscle, with consequences for regenerative capacity under obesity. *SFRP4* was the only shared upregulated gene. *SFRP4* antagonizes Wnt ligands and suppresses Wnt/β-catenin signaling, and Wnt signaling in skeletal muscle promotes satellite cell activation and myogenic differentiation [30]. *SFRP4* upregulation may therefore dampen muscle regenerative capacity. Beyond muscle, *SFRP4* is elevated in obesity-related dysglycemia and correlates with insulin resistance [31], and has been shown to impair hepatic insulin action and promote lipogenesis [32]. Together, these three genes implicate perturbations in insulin/Akt signaling (*PHLDA3*), MAPK-mediated regeneration (*CNKSR2*), and Wnt-dependent muscle maintenance (*SFRP4*) in obese skeletal muscle.

Among all DEGs, *SCD* was the most strongly upregulated gene (mean log2FC = 2.513). *SCD* encodes stearoyl-CoA desaturase, which catalyzes the rate-limiting step in the conversion of saturated to monounsaturated fatty acids, thereby regulating membrane lipid composition, triglyceride synthesis, and lipid partitioning in skeletal muscle [33]. Elevated *SCD* expression has been consistently observed in obese human muscle. Hulver et al. demonstrated that primary myocytes derived from extremely obese individuals retain elevated *SCD* activity and a lipogenic phenotype even in culture, suggesting that *SCD* upregulation reflects an intrinsic metabolic reprogramming of muscle cells rather than a transient response to the systemic environment [34]. The pronounced *SCD* upregulation in our meta-analysis across four independent cohorts reinforces that lipid metabolic remodeling is a robust transcriptional feature of obese skeletal muscle, likely contributing to intramyocellular lipid accumulation and impaired insulin sensitivity. In contrast, *IGFN1* was the most strongly downregulated gene (mean log2FC = −2.427). *IGFN1* is a large structural protein highly expressed in skeletal muscle that has been reported to decline with aging and sarcopenia [35]. Functionally, Li et al. showed that shRNA-mediated knockdown of *IGFN1* broadly impairs myoblast fusion and differentiation, indicating that *IGFN1* is required for normal myofiber formation [36]. Its marked downregulation in obese muscle across studies suggests that obesity may compromise the structural and regenerative machinery necessary for muscle maintenance, independent of age-related sarcopenia. Together, the upregulation of *SCD* and downregulation of *IGFN1* point to a dual transcriptional shift in obese skeletal muscle: enhanced lipid metabolic activity coupled with diminished myogenic and structural capacity. This pattern is consistent with the clinical observation that obese individuals often exhibit reduced muscle quality despite preserved or increased muscle mass.

Functional enrichment analysis was performed separately for upregulated and downregulated DEGs to capture direction-specific biological signals. Among downregulated genes, ribosomal and translation-related terms predominated. Cytoplasmic translation was the top GO biological process term (77 genes, adjusted *p* = 2.69 × 10^−38^), and the ribosome was the leading KEGG pathway (96 DEGs, adjusted *p* = 1.87 × 10^−21^). Das et al. similarly reported downregulation of ribosomal function genes in skeletal muscle of individuals with elevated BMI across two independent ethnic cohorts [37]. At the functional level, obesity has been associated with impaired myofibrillar protein synthesis following feeding and resistance exercise, a phenomenon termed anabolic resistance [38]. The transcriptional suppression of ribosomal genes observed here may contribute to this impairment.

Oxidative phosphorylation (50 genes, adjusted *p* = 3.09 × 10^−16^), cellular respiration (73 genes, adjusted *p* = 3.59 × 10^−21^), and ATP biosynthesis (34 genes, adjusted *p* = 1.21 × 10^−10^) were also strongly enriched among downregulated genes. Coordinated transcriptional suppression of oxidative phosphorylation gene sets in insulin-resistant skeletal muscle was first reported by Mootha et al. and Patti et al. [39,40], and has been corroborated by the evidence of reduced subsarcolemmal mitochondrial content in obese individuals [5]. The non-alcoholic fatty liver disease pathway was additionally enriched (52 DEGs, adjusted *p* = 2.17 × 10^−5^), likely reflecting shared mitochondrial and lipid metabolism gene sets between the liver and skeletal muscle.

Among upregulated genes, extracellular matrix organization was the most significant GO term (51 genes, adjusted *p* = 1.38 × 10^−10^), and focal adhesion (47 DEGs, adjusted *p* = 3.74 × 10^−2^) and integrin signaling (39 DEGs, adjusted *p* = 1.22 × 10^−3^) were enriched at the pathway level. Increased collagen deposition has been documented in skeletal muscle of obese and insulin-resistant individuals [41], and short-term overfeeding in healthy subjects is sufficient to upregulate collagen and matrix metalloproteinase expression alongside focal adhesion pathway activation [42]. In animal models, collagen–integrin signaling has been shown to directly mediate high-fat-diet-induced muscle insulin resistance [43]. These findings suggest that ECM remodeling and fibrosis may be a common transcriptional feature of obese skeletal muscle.

Several limitations should be acknowledged. First, individual-level covariate information was available for only two of the four datasets. The remaining two studies were modeled with group alone, leaving potential residual confounding by demographic factors. Second, the overall sample size was modest (*n* = 52; 23 normal weight, 29 obese), limiting statistical power for small effect sizes and increasing uncertainty near the significance threshold. Third, with only four studies, I^2^ confidence intervals are wide, and gene-level heterogeneity estimates should be interpreted with caution. Finally, technical and design differences across studies—including sequencing platform, library preparation protocol, read depth, and cohort selection criteria—may introduce systematic variability that cannot be fully resolved by reprocessing through a standardized pipeline; larger cohorts with prospective designs will be needed to address this limitation.

## 4. Materials and Methods

### 4.1. Data Sources and Search Methodology

A systematic search was conducted in PubMed and the Gene Expression Omnibus (GEO) databases, covering all available records up to January 2024. In PubMed, the search strategy involved a combination of Medical Subject Headings (Mesh) terms, specifically ‘Obesity’ and ‘Muscle, Skeletal,’ along with their respective subheadings and synonyms. For GEO, the search utilized the terms ‘Obesity’ and ‘Skeletal Muscle’.

### 4.2. Study Inclusion and Exclusion Criteria

The articles were independently selected by two reviewers. Disagreements were resolved by a third reviewer.

Inclusion criteria: (1) use of RNA-seq, (2) diagnosis of obesity based on WHO criteria, (3) the presence of a control group consisting of individuals with normal body weight, and (4) the inclusion of tissue samples obtained from the human vastus lateralis muscle.

Exclusion criteria: (1) samples derived from metabolic surgery, acute exercise, or other interfering factors, (2) repetitive studies, (3) review articles, (4) studies lacking RNA-seq raw count matrices in GEO.

### 4.3. Differential Expression Analysis, Meta-Analysis, and Functional Enrichment

A total of four RNA-seq studies were included in the final analysis, and their details are presented in Table 1. The demographic characteristics of the study participants are summarized in Table 2. The corresponding RNA-seq raw count matrices were obtained from NCBI-generated RNA-seq count data in the GEO database. NCBI employed HISAT2 to align the SRA runs to the genome assembly GCA_000001405.15. Subsequently, runs that achieved an alignment rate of 50% or higher were processed using Subread featureCounts. Gene annotation was performed using Homo sapiens Annotation Release 109.20190905. Finally, the SRR raw count files were converted into GEO Series raw count matrices. In cases where multiple SRA runs were associated with a single GEO sample, the raw counts were summed across runs.

Differentially expressed genes (DEGs) were identified independently for each study using DESeq2 (v1.40.2). Genes with low expression were filtered prior to analysis, retaining only those with counts ≥ 10 in at least as many samples as the smaller group size. To account for potential confounding, covariates were incorporated into the DESeq2 design formula when individual-level demographic data were available in GEO. Specifically, age was included as a covariate for GSE137631, in which all participants were female, and both age and sex were included for GSE231509. For GSE185957 and GSE196387, individual-level covariate information was not available in GEO, and a group-only design (~group) was used.

For cross-study integration, we used a direction-aware weighted Stouffer (inverse normal) approach [44]. Only genes that were available across all four studies after DESeq2 processing were carried forward. In each study, DESeq2 Wald statistics were taken with their sign preserved and combined across studies using weights proportional to the square root of the number of included biological replicates. The resulting *p*-values were adjusted using the Benjamini–Hochberg method, and genes with adjusted *p*-values < 0.05 were considered significant in the meta-analysis. Genes showing opposite effect directions across studies (based on the sign of log2 fold changes/Wald statistics) were marked as direction-inconsistent; they were not removed from the main analysis.

To assess between-study heterogeneity, a gene-level random-effects meta-analysis of log2 fold changes was conducted using the “metafor” R package (version 4.4-0) [45] with restricted maximum likelihood (REML) estimation, and the I-squared statistic was computed.

A Venn diagram was generated using the “VennDiagram” R package (Version 1.7.3) to illustrate the overlap of DEGs across individual studies and the meta-analysis. Gene Ontology (GO) enrichment and KEGG pathway analyses were performed using the “clusterProfiler” R package (Version 4.8.3). All DEGs from the meta-analysis were mapped to terms in the GO and KEGG databases. A threshold of adjusted *p*-value and q-value < 0.05 was applied to identify significantly enriched terms. GO enrichment analysis categorized the DEGs into three subgroups: biological process (BP), cellular component (CC), and molecular function (MF). All statistical analyses were performed in R (version 4.3.3; R Foundation for Statistical Computing, Vienna, Austria).

## 5. Conclusions

Our study is the first meta-analysis of RNA-seq studies conducted on skeletal muscle in individuals with obesity. Our meta-analysis has successfully identified genes associated with skeletal muscle metabolism in individuals with obesity, encompassing both previously reported and newly discovered genes. For the newly discovered genes, the relationship between these genes and skeletal muscle metabolism in obesity has not been investigated. Further investigation is required to explore the functions of these DEGs and to examine their roles in skeletal muscle metabolism in obesity.

## Figures and Tables

**Table 1:** GEO datasets included in meta-analysis.

Study	Dataset	Platform	Source	Samples	
				Included	Excluded
Study_1	GSE137631	Illumina NextSeq 500 (San Diego, CA, USA)	vastus lateralis muscle	6 NW ^1^ + 12 OB ^2^	6 RYGB ^3^ + 6 Exercise ^4^
Study_2	GSE185957	Illumina HiSeq 4000	vastus lateralis muscle	4 NW ^1^ + 4 OB ^2^	8 Exercise ^5^
Study_3	GSE231509	Illumina NextSeq 550	vastus lateralis muscle	7 NW ^1^ + 7 OB ^2^	14 T2DM ^6^ + 14 MMTT ^7^
Study_4	GSE196387	Illumina NovaSeq 6000	vastus lateralis muscle	6 NW ^1^ + 6 OB ^2^	6 T2DM

Notes: ^1^, normal weight; ^2^, simple obese; ^3^, Roux-en-Y gastric bypass surgery; ^4^, received exercise training; ^5^, after an acute exercise; ^6^, type 2 diabetes mellitus; ^7^, mixed meal tolerance test.

**Table 2:** Demographic characteristics of study participants.

Study	Non-Obese				Obese			
	*n*	Sex (F/M)	Age (Years)	BMI (kg/m^2^)	*n*	Sex (F/M)	Age (Years)	BMI (kg/m^2^)
GSE137631	6	6 F/0 M	36.7 ± 5.4	22.7 ± 1.4	12	12 F/0 M	38.4 ± 5.5	47.9 ± 6.1
GSE185957	4	2 F/2 M	NR	24 ± 2	4	4 F/0 M	NR	37 ± 3
GSE231509	7	3 F/4 M	42.7 ± 9.0	22.3 ± 2.2	7	4 F/3 M	37.1 ± 14.1	44.9 ± 7.4
GSE196387	6	0 F/6 M	34 ± 10	22.9 ± 2.0	6	5 F/1 M	46 ± 7	36.2 ± 6.7

NR, not reported. Age and BMI are presented as mean ± SD.

## Data Availability

The original contributions presented in this study are included in the article/Supplementary Materials. Further inquiries can be directed to the corresponding author.

## Supplementary Materials

The following supporting information can be downloaded at https://www.mdpi.com/article/10.3390/ijms27062677/s1.
