# Supplementary figures and images for: Meta-Analysis of RNA-Seq Data Identifies Differentially Expressed Genes in Skeletal Muscle Between Obese and Normal Weight Individuals
Source: Int J Mol Sci. 2026 Mar 15;27(6):2677. doi: 10.3390/ijms27062677 (PMC13026894; doi:10.3390/ijms27062677)

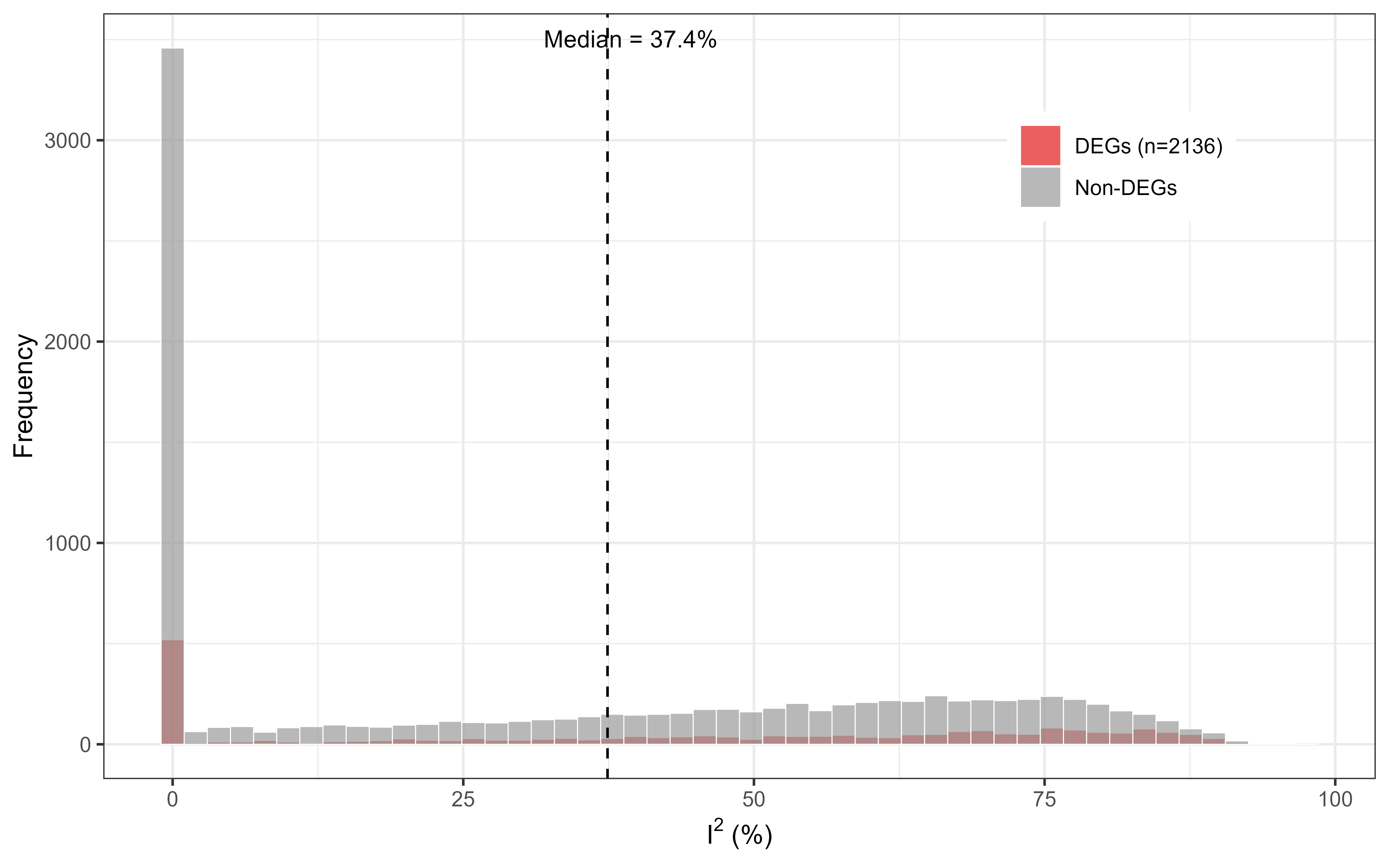

Supplement: Supplementary file 1 [file ijms-27-02677-s001.zip › FigureS1_I2_distribution.png]
